# Recurrence of retroperitoneal localized perivascular epithelioid cell tumor two years after initial diagnosis: case report

**DOI:** 10.1590/1516-3180.2017.0120050717

**Published:** 2018-01-15

**Authors:** Yasemin Benderli Cihan, Engin Kut, Ali Koç

**Affiliations:** I MD. Radiation Oncologist, Department of Radiation Oncology, Kayseri Egitimve Arastirma Hastanesi, Kayseri, Turkey.; II MD. Medical Oncologist and Internal Medicine Specialist, Department of Medical Oncology, Kayseri Egitimve Arastirma Hastanesi, Kayseri, Turkey.; III MD. Radiologist, Department of Radiology, Kayseri Egitimve Arastirma Hastanesi, Kayseri, Turkey.

**Keywords:** Perivascular epithelioid cell neoplasms, Retroperitoneal neoplasms, Antineoplastic protocols

## Abstract

**CONTEXT:**

Perivascular epithelioid cell tumors (PEComas) are rare mesenchymal tumors. Adjuvant radiotherapy and/or chemotherapy are administered according to the patient’s clinical characteristics.

**CASE REPORT:**

A 42-year-old female patient was operated to treat a retroperitoneal mass. The diagnosis was established as PEComa with benign behavior. Two years after the diagnosis, chest and abdominal computed tomography scans showed intra-abdominal recurrence and lymphangioleiomyomatosis in the lung. Treatment with everolimus was started. The disease stabilized in the third month of treatment, according to the response evaluation criteria in solid tumors.

**CONCLUSION:**

PEComas are tumors with unpredictable behavior. Therefore, these patients require long-term follow-up, even in cases of correct diagnosis and benign PEComa.

## INTRODUCTION

Perivascular epithelioid cell tumors (PEComas) are mesenchymal tumors consisting of perivascular epithelioid cells that can be histologically and immunohistochemically distinguished and are rarely seen. In general, these tumors are seen in women aged around 50 years. The most common location is the retroperitoneum.^1^^,^^2^ However, these tumors have been reported in several anatomical regions, including the visceral organs, soft tissues, prostate and broad ligament. Tumors located in the retroperitoneum grow insidiously and manifest as huge lesions. In 2002, the World Health Organization (WHO) reclassified tumors involving PEComas such that they included angiomyolipoma, clear-cell “sugar” tumor, lymphangioleiomyomatosis, clear-cell myomelanocytic tumor of the ligamentum teres/falciform ligament and PEComa not otherwise specified. Primary PEComas may be benign, malignant or otherwise specified with unknown potential for malignancy. The majority of these tumors are benign with a good prognosis.^1^^,^^2^^,^^3^

In this report, we present a patient who was operated to treat a retroperitoneal mass that was diagnosed as a benign PEComa, and who developed abdominal recurrence and pulmonary lymphangioleiomyomatosis.

## CASE REPORT

A 42-year-old female patient was seen in October 2014, with complaints of abdominal pain and nausea/vomiting that had been occurring for the last three months. In her medical history, she had been using colchicines for 15 years with the diagnosis of familial Mediterranean fever (FMF).

**Figure 1:**
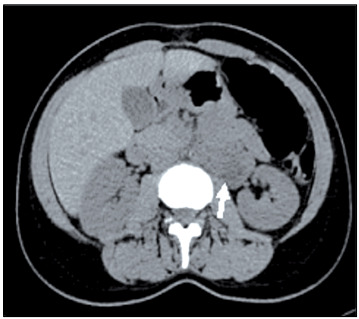
Axial abdominal computed tomography image showing hypodense mass lesion in the left anterior pararenal space with well-defined borders.

Abdominal ultrasonography (USG) showed a lesion of cystic appearance that measured 8 cm x 3 cm, starting from the lower pole of the left kidney, which was located anteriorly to the iliopsoas muscle. Computed tomography (CT) scans showed a hypodense lesion that measured 5.5 cm x 9.0 cm x 3.0 cm and was located retroperitoneally. It had a smooth outline, started from the level of the left renal artery and expanded from anterior to the iliopsoas muscle towards an inferior position. The backgrounds of the lesion and iliopsoas muscle were not clearly visible (Figure 1).

The patient underwent local mass excision. Histopathological examination showed PEComa (lymphangioleiomyomatosis). No atypia or necrosis was observed. Onemitosis was present and Ki67 proliferation in 2% of cells was observed. The diagnosis was established as PEComa with benign behavior. Chemotherapy and radiotherapy were not recommended for this patient.

The patient returned with complaints of shortness of breath and back pain two years after the diagnosis and was diagnosed as presenting pneumothorax. Chest and abdominal CT scans showed intra-abdominal recurrence and lymphangioleiomyomatosis in the lungs. The pneumothorax was treated by means of chest tube placement.

The patient was also evaluated for a biopsy. However, the intra-abdominal lesions were small and difficult to biopsy. Treatment with everolimus at 10 mg/day was started. This was well tolerated, except that a grade 1 acneiform rash occurred on the patient’s back, which was relieved by means of topical steroid.

**Figure 2:**
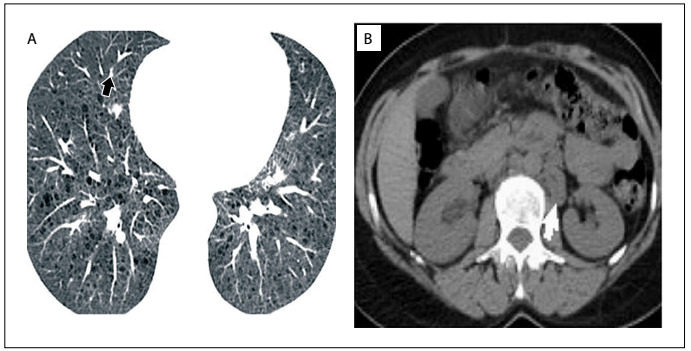
A) Axial abdominal computed tomography image showing residual tumor (arrow). B) Lung computed tomography image showing tiny diffuse parenchymal cystic air spaces.

Positron emission tomography (PET)-CT indicated minimal regression at the third month of treatment (Figure 2A and B). However, the disease had become stable according to the response evaluation criteria in solid tumors (RECIST).^4^ Everolimus has been continued for nine months.

## DISCUSSION

No optimal treatment approach has been standardized for PEComas. The standard treatment is surgery plus chemotherapy. It is important to reach negative surgical margins. Chemotherapy forms the basis for treatment and can be combined with radiotherapy. Recently, developments towards targeted treatments have shown promise.^2^^,^^3^^,^^4^^,^^5^

PEComas show evidence of mammalian target of rapamycin (mTOR) activation, but the mechanisms for its activation remain unclear. Tuberous sclerosis complex (TSC) 1 or 2 tumor suppressor genes regulate mTOR kinase. Defects in mTOR kinase lead to an increased signal pathway, transduction and cell proliferation. mTOR inhibitors, such as everolimus block this signal pathway and decrease cell proliferation. There have been several reports of treatment of metastatic PEComa with mTOR inhibitors. In a case series, mTOR inhibitors were reported to be reliable and effective, especially for treating unresectable recurrent tumors and cases with distant metastases.^3^

Because the number of reported cases is limited (Table 1), there are no consistent criteria for diagnosing and treating benign or malignant PEComas. Aggressive progression is observed in malignant cases presenting two or more of the following criteria: marked atypia and mitosis, vascular infiltration, infiltrative growth, high nuclear grade, tumor diameter greater than 5 cm, high mitotic activity (> 1 mitotic figure/50 high power fields), tumor necrosis and increased cellularity. Despite postoperative radiotherapy, chemotherapy and/or immunotherapy (which may be implemented separately or in combination), the prognosis is poor in cases of these tumors with a malignant course. There was only one criterion for malignancy in our patient (tumor diameter > 5 cm). This case was then considered to be one of benign PEComa, since no other criterion was found.^2^^,^^3^

**Table 1:** Results from search of the literature

Database	Search strategy	Results	
		Found	Related
MEDLINE (via PubMed, July 14, 2017)	#1 (“Perivascular epithelioid cell neoplasms”[MeSH]) #2 (“Retroperitoneal neoplasms”[MeSH]) #3 #1 AND #2 Filters: Case Reports	214	9
LILACS (via Bireme)	#1 mh:(Perivascular epithelioid cell neoplasms) #2 mh:(Retroperitoneal neoplasms) #3 #1 AND #2	0	--

To the best of our knowledge, only 20 cases of retroperitoneal PEComa have been reported in the literature so far,^1^^,^^6^^,^^7^^,^^8^^,^^9^^,^^10^ some of them commented below. Our case is the only one in which there was local recurrence and pulmonary metastasis, two years after the initial diagnosis of benign PEComa.

Pata et al.^1^ performed total resection in a 66-year-old female patient with synchronous diffuse pulmonary lymphangioleiomyomatosis with a large retroperitoneal PEComa. Their patient was followed up without adjuvant therapy. They did not detect any local recurrence or metastasis at the end of the first year.^1^ Benson et al. conducted a retrospective study on ten cases and observed partial response in five patients and stable disease in one patient.^4^ Gennatas et al. obtained a significant response over the course of the follow-up on a patient who received 10 mg of everolimus for 10 months and subsequently reached survival of 37 months after surgery.^5^ Wagner et al. reported a case of recurrent retroperitoneal PEComa and started administration of another mTOR inhibitor, sirolimus (8 mg/day). At the end of the first year, the tumor had regressed almost completely, while at the end of the 16^th^ month they reported that both the treatment and the response remained the same.^3^ In our case, we achieved minimal regression with everolimus.

## CONCLUSION

PEComas located retroperitoneally are rarely seen. These lesions are generally confused with stromal tumors. PEComas are tumors with unpredictable behavior. Therefore, these patients require long-term follow-up, even in cases of correct diagnosis and benign PEComa.
